# Assessing the association between unstimulated whole salivary flow rate (UWSFR) and oral health status among healthy adult subjects: A cross-sectional study

**DOI:** 10.4317/medoral.22245

**Published:** 2018-06-21

**Authors:** Majdy Idrees, Mohammad-Zakaria Nassani, Omar Kujan

**Affiliations:** 1 Private Dental Sector, Manama, Kingdom of Bahrain; 2 Department of Prosthetic Dental Sciences, AlFarabi College for Dentistry and Nursing, Riyadh, Saudi Arabia; 3 UWA Dental School, University of Western Australia, Nedlands WA6009, Australia

## Abstract

**Background:**

This study aimed to test the association between the unstimulated whole salivary flow rate (UWSFR) and the oral health status represented by dental and gingival status among healthy adult subjects.

**Material and Methods:**

This work was a cross-sectional study of patients attending the undergraduate dental clinics at AlFarabi College for Dentistry and Nursing, Riyadh, Saudi Arabia. The study population consisted of 502 systemically healthy adults aged 18–35 years. UWSFR was collected for all study participants and expressed as ml/min. Oral health status was estimated using the Community Periodontal Index (CPI) and the Oral Hygiene Index-Simplified (OHI-S). The number of decayed teeth and the number of available teeth were also calculated to evaluate dental status.

**Results:**

The mean UWSFR was 0.42 (±0.3) ml/min, and the male participants significantly had more UWSFR than the females. UWSFR was significantly affected by CPI, OHI-S, body mass index (BMI) and gender as indicated in the univariate analysis. However, multiple regression analysis revealed that only gender was a significant predictor of UWSFR. The male subjects were shown to have a higher average of 0.133 ml/min than the females.

**Conclusions:**

High BMI scores, moderate-to-severe gingivitis and low level of oral hygiene increased UWSFR. However, further longitudinal studies are recommended to test the role of salivary cytokine levels to validate the exact association between the UWSFR and the oral health status.

** Key words:**Cross-sectional study, Saliva, oral health, CPI, OHI-S.

## Introduction

The role of saliva is critical in preserving and maintaining the health of oral mucosal tissues (1). Among the many functions of saliva, saliva has a principal role in the protection and maintenance of oral mucosa (2). Saliva contains antimicrobial components that protect against dental caries and oral infections (3). It has a buffering capacity to neutralise the pH of the oral cavity and prevent teeth demineralisation. Furthermore, it contains many electrolytes that aid in teeth mineralisation (3). Saliva has been used as a non-invasive diagnostic tool to detect and evaluate the severity of several systemic and oral conditions (4). For example, saliva is widely used to assess endocrine functions, monitor drug concentrations and measure antibodies and antigens (4) .

The role of saliva in protecting against dental caries is well studied and documented. Much evidence reports that increased salivary flow is associated with increased clearance rate and buffering capacity, thus reducing the caries risk significantly (5). Consequently, any disorder that leads to salivary hypo-secretion causes severe caries and mucosal inflammation (6).

Interestingly, the association between periodontal conditions and salivary flow rate remains uncertain as that reported with dental caries. Studies demonstrated that the unstimulated whole salivary flow rate (UWSFR) has significantly low values in patients with periodontal diseases (7). By contrast, other studies revealed a high UWSFR in subjects diagnosed with periodontal diseases (8). To add more confusion to the argument, others denied any association between these variables (9). Therefore, the current study aimed to examine the association between the UWSFR and the oral health status as represented by the Community Periodontal Index (CPI), Oral Hygiene Index-Simplified (OHI-S), number of decayed teeth and number of teeth present in a cohort of a young healthy adult population.

## Material and Methods

-Study design

This cross-sectional study was prepared in accordance with the STROBE statement and conducted at the undergraduate dental clinics of AlFarabi College for Dentistry and Nursing in Riyadh, Saudi Arabia, between June and December 2015. Participants were randomly selected from patients visiting the undergraduate dental clinics who typically met our inclusion criteria. All study measures were ethically approved by the Institutional Review Board of AlFarabi College and were in accordance with the principles of the Helsinki Declaration (IRB: OM;0219). All participants were asked to sign a consent form after the objectives of the study were presented.

A full medical history including a detailed medication history for the last six months was taken from each participant to validate his/her eligibility for study enrolment. All dental examination procedures were undertaken on a standard dental unit by an calibrated experienced periodontist using a sterile dental kit with the standard white headlight. Intra-examiner reliabity of the oral health and periodontal examinations was undertaken on a group of 10 subjects and has shown a high score of Cohen k exceeding value of 0.9.

-Inclusion criteria

1. Healthy subjects (were those participants without systemic diseases neither controlled nor non-controlled)

2. Adults aged 18–35 years

-Exclusion criteria 

1. Patients with clinical attachment loss or periodontitis

2. Patients with any chronic medical or systemic disease, are currently under medications or have received any medications in the last six months prior to the visit.

3. Smokers

4. Patients wearing fixed or removable prosthesis 

5. Patients who are currently undergoing orthodontic treatment

6. Female patients who are pregnant, lactating, or taking oral contraceptives

7. Patients who did not consent 

-Saliva collection

The saliva collection procedure was undertaken in the morning prior to any dental examination. All participants were asked to avoid eating, drinking, chewing gum and brushing their teeth 1 hour before the procedure to exclude any external factors that could affect the amount of saliva secretion (10,11). The subjects were asked to relax for 5 min and to swallow all saliva present in their mouth before saliva collection. Following previous publications (11-14), unstimulated whole saliva was then collected for 5 min in a graduated tube to be measured by volume and expressed as ml/min.

The participants were classified according to the amount of UWSFR into three groups(1).

• Very low UWSFR ≤ 0.1 ml/min

• Low UWSFR 0.11–0.25 ml/min

• Normal UWSFR > 0.25 ml/min

Body mass index (BMI) calculation

BMI was calculated for all subjects to validate the effect of weight on salivary flow. BMI is defined as the body weight divided by the square of the body height and is universally expressed as kg/m2 (15). The weight and height of the study subjects were measured by a calibrated digital scale (Beurer, Germany). The subjects were classified according to their BMI into four groups: underweight subjects (BMI < 18.5 kg/m2), normal weight subjects (BMI 18.5–25 kg/m2), overweigh subjects (BMI 25–30 kg/m2) and obese subjects (BMI > 30 kg/m2) .

-Oral health evaluation 

The CPI and OHI-S were used for all participants to evaluate their gingival and oral hygiene status (16,17). The number of decayed teeth and the total number of presenting teeth were calculated. The World Health Organization (WHO) criteria for defining and diagnosing the dental caries were considered in this study to calculate the number of decayed teeth of each patient (17).

The CPI is recommended by the WHO as a reputable index for epidemiological periodontal studies (17,18). To determine the value for each participant, the teeth were divided into six sextants: four posterior areas and two anterior areas. The participants were excluded if they had less than two teeth in each sextant. Ten index teeth were examined in each participant ( Table 1). Each tooth is given a score of 0 to 2 as follows: healthy gingiva (score 0), bleeding while probing (score 1) and calculus detection with probing depth less than 4 mm (score 2). The mean of the CPI for all 10 index teeth was calculated for each participant.

**Table 1:**
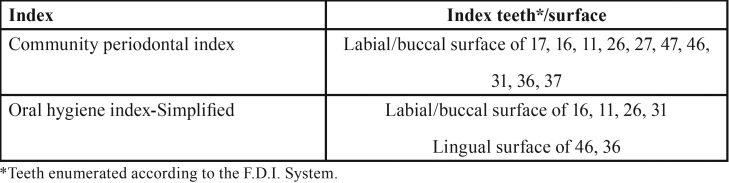
Index teeth for CPI and OHI-S.

The OHI-S measures the amount of plaque and calculus accumulation on tooth surfaces instead of the presence/severity of gingival inflammation. The OHI-S represents the sum of the mean of two indices: debris index and calculus index ( Table 2) (16). Similar to CPI, specific index teeth are considered representative of other teeth ( Table 1). According to the individual value of OHI-S, the subjects were classified into three groups: good (0–1.2), fair (1.3–3.0) and poor (3.1–6.0) (16).

**Table 2:**
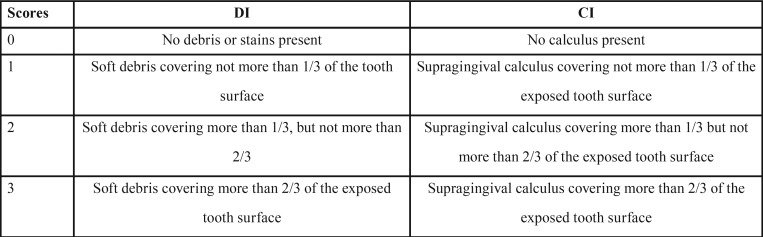
Criteria of measuring debris index (DI) and calculus index (CI).

Statistical analysis

Data were analysed using the Statistical Package for Social Sciences software version 22.0. Data on the numerical variables were expressed as mean and standard deviation, and the categorical variables were described as frequency and percentage. One-way ANOVA and Spearman’s correlation test were used to examine the differences among the variables. Post hoc Tukey’s multiple comparisons were used following the one-way ANOVA to determine which pairs of means were statistically significant. Variables with *P* ≤ 0.20 in the initial analyses were included into the multiple stepwise regression model as independent variables to validate their role as predictors. A *p*-value of < 0.05 was considered statistically significant.

## Results

-Subjects 

Out of the 574 subjects that were initially interviewed, 502 were eligible to be included in the study, with 192 (38.2%) being males and 310 (61.8%) being females. The age range was 18–35 years. The mean age of the participants was 24.2 (±4.9) years, with the mean age of the males being 26.5 (±4.8) years and that of the females being 22.8 (±4.5) years. The mean values of BMI, CPI and OHI-S were significantly higher among the male subjects than among the female subjects, and the number of remaining teeth was significantly lower in the males than in the females. No significant relation was reported between gender and number of decayed teeth ( Table 3).

**Table 3:**
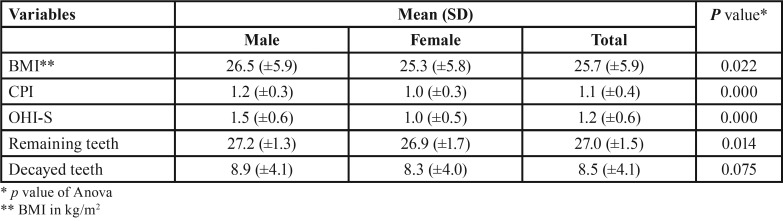
Association between subject’s gender and other variables.

-UWSFR

The mean UWSFR was 0.42 (±0.3) ml/min, and the median flow rate was 0.32 ml/min (range 0.1–2 ml/min). Sixty-five subjects (12.9%) had a very low UWSFR of 0.1 ml/min, and 138 subjects (27.5%) had a low UWSFR of 0.11–0.25 ml/min. The remaining 299 subjects (59.6%) had a normal UWSFR of greater than 0.25 ml/min.

-Associations between patient variables and UWSFR

**Figure 1:**
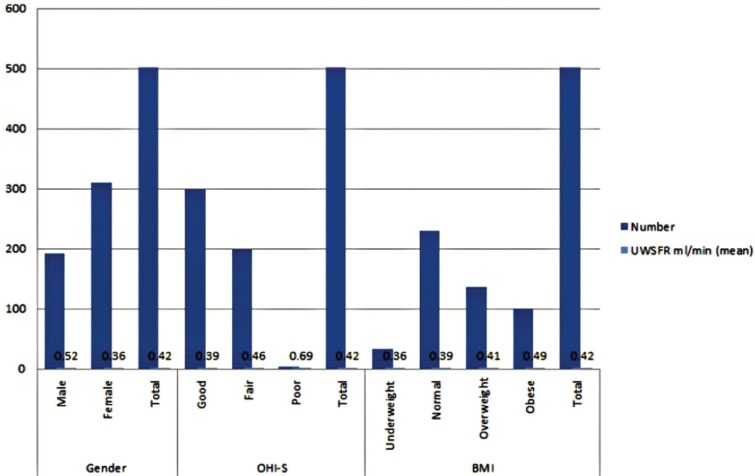
Patients variables and its relation to UWSFR.

The mean of the UWSFR was significantly higher among the males than among the females (Fig. 1). The UWSFR was significantly related to BMI (*p* = 0.039), as determined by the one-way ANOVA. The major and statistically significant difference revealed by Tukey’s post hoc comparisons was found among obese subjects and those who had normal weight (*p*=0. 042). The differences between the other groups were not statistically significant (Fig. 1).

Another significant association was reported between the OHI-S group and UWSFR (*p*=0.008). Tukey’s post hoc analysis revealed that the increase in UWSFR from good to fair oral hygiene was statistically significant (*p* = 0.026), but no other group differences were statistically significant (Fig. 1).

A Spearman’s rank–order correlation was run to assess the relationship between the mean of the CPI, the number of available teeth and the number of decayed teeth with the amount of UWSFR. A positive correlation was observed between the CPI and UWSFR (RS (98) = 0.18, *p* < 0.000). No significant correlation was found between the UWSFR and the number of available teeth (RS (98) = 0.006, *p* = 0.891) or the number of decayed teeth (RS (98) = -0.027, *p* = 0.551).

-Multivariate analysis

Multivariate regression analysis was run to validate the role of gender, CPI, BMI and OHI-S as predictors of the UWSFR. When the effect of other factors was controlled, gender was found to be the most important predictor. The predicted UWSFR for the males was 0.075–0.19 ml/min greater than that for the females (average 0.133 ml/min, *P* = 0.000).

## Discussion

The present study was designed to avoid the flaws found in previous published papers that evaluated the association between the oral health status and the salivary flow. The literatures that assessed the relation between UWSFR and periodontal health among medically fit subjects were not consensus. Therefore, we applied strict inclusion criteria to minimise the role of any confounders that could affect either the saliva flow rate or the oral health status.

The notion of age-related decline in saliva output has been postulated for several decades (19), but no specific age for this decline has been identified yet. Sawair *et al.* (13) revealed that the UWSFR decreased by 0.002–0.006 ml/min for every one year increase in age. Our inclusion criteria excluded any subject aged over 35 years to rule out the aging effect on the function of the salivary glands. Smokers were also excluded in the present study to avoid the damaging effect of smoking on saliva secretion and oral hygiene status (20).

This study aimed to examine the association between the oral health status and the unstimulated salivary flow rate. Thus, we concentrated on the mean of the CPI instead of on the number of sextants with periodontal diseases, which is important in prevalence studies. Moreover, the mean number of sextants affected by each CPI score gives a better representation of gum status at the population level (17). Our criteria were limited to participants with healthy gums or with gingivitis. Thus, we excluded subjects diagnosed with the periodontal condition of periodontitis, which represents a severe form of periodontal disease accompanied by attachment loss (21). Although periodontitis is considered a consequence of gingivitis, they are different conditions (22). Furthermore, periodontitis is considered an age-related condition that affects people as a result of cumulative destruction (23). These variations between gingivitis and periodontitis could influence the mechanism of saliva secretion at the cellular or immunity level, which is beyond the aim of this study.

Gender played a significant role in the present study. The mean CPI and OHI-S was significantly higher among the males than among the females, consistent with the many studies that revealed a better oral hygiene and periodontal status among the females than among the males (24-26). Subjects with higher BMI were found to have larger salivary glands (27) and thus a higher salivary flow rate. Thus, our result that the obese subjects significantly had greater UWSFR than the normal weight subjects was justified.

The mean of the UWSFR is considerably varied among different studies from different countries. However, these variations may be related to the differences in ethnic characteristics and study designs. The mean of the UWSFR for Saudi subjects as measured in this study is comparable with that reported in similar studies in Jordan (13), Spain (12) and the United Kingdom (28). However, the effect of the number of remaining teeth on the UWSFR is not clearly understood (13). Our results demonstrated no association between the number of teeth and the UWSFR in contrast to a previous study that reported a significant correlation between the number of teeth and the UWSFR among older subjects aged 50 years and above (29). This discrepancy can be attributed more to the aging process than to the actual number of remaining teeth. Unsurprisingly, no significant association was reported in this study between the number of decayed teeth and the UWSFR, as the lowest UWSFR in our study was 0.1 ml/min. Apparently, the relationship between dental caries and saliva was confirmed among patients with Sjogren’s syndrome or xerostomia, in which the stimulated saliva flow is less than 0.1 ml/min (30).

Although the role of saliva as a protective measure against oral pathogens is well studied, the association between salivary flow and periodontal conditions is still debatable. Our results revealed that saliva secretion increases significantly with the increased severity of gingival disease and with poor oral hygiene. This finding is consistent with those of previous works that showed a similar relation (8,10). Zulkarnain *et al.* reported that subjects with periodontal diseases had about 8 times more UWSFR compared to subjects without periodontal diseases (8). Likewise, Rajesh *et al.* revealed that increased salivary flow rate was directly proportional with developing periodontitis (10). On the contrary, our result is also contradicted by those of other studies that found a negative or no association between the periodontal status and the resting salivary flow rate (7,9). Their findings were limited by confounding variables, such as age, small sample size and unrestricted inclusion and exclusion criteria.

Note that there is one potential explanation for the relation between the UWSFR and the periodontal status, which is related to the biology of the sympathetic nervous system (SNS) in the salivary glands. The SNS stimulates salivary secretion to enhance respiration or to react to a harmful situation, such as the presence of infection or inflammation . Consequently, the high UWSFR found in our study among subjects with higher CPI and OHI-S could be due to the inflammatory activity of gingival tissues triggering the SNS related to the salivary glands to secrete more saliva. Nevertheless, future research is warranted to test the hypothesis on the role of gingival status and its associated inflammatory cytokine levels on saliva production.

We used the multivariate statistical analysis to control the significant independent factors affecting the UWSFR. Our findings showed that gender is only considered as a significant independent predictor of the UWSFR. Fenoll-Palomares *et al.* (27) attributed these differences between gender groups to the anatomical differences between males and females, as males tend to have greater glandular size than females. Similar to those of previous studies, our findings showed a significantly higher UWSFR among males than among females (12,28,29).

## Conclusions

Our findings support that higher BMI scores, moderate-to-severe gingivitis and low level of oral hygiene are associated with an increased UWSFR. However, further longitudinal studies are recommended to test the role of salivary cytokine levels to validate the exact association between the UWSFR and the oral health status.

## References

[1] de Almeida Pdel V, Gregio AM, Machado MA, de Lima AA, Azevedo LR (2008). Saliva composition and functions: A comprehensive review. J Contemp Dent Pract.

[2] Hall HD (1993). Protective and maintenance functions of human saliva. Quintessence Int.

[3] Benn AM, Thomson WM (2014). Saliva: An overview. N Z Dent J.

[4] Tumilasci OR, Arqueros MC, Ostuni MA, el Tamer E, Houssay AB (1996). Thyrotropin receptor antibodies in parotid saliva. J Endocrinol Invest.

[5] Stookey GK (2008). The effect of saliva on dental caries. J Am Dent Assoc.

[6] van der Reijden WA, van der Kwaak JS, Veerman EC, Nieuw Amerongen AV (1996). Analysis of the concentration and output of whole salivary constituents in patients with sjogren's syndrome. Eur J Oral Sci.

[7] Marton K, Madlena M, Banoczy J, Varga G, Fejerdy P, Sreebny LM (2008). Unstimulated whole saliva flow rate in relation to sicca symptoms in hungary. Oral Dis.

[8] Rajesh KS, Zareena, Hegde S, Arun Kumar MS (2015). Assessment of salivary calcium, phosphate, magnesium, ph, and flow rate in healthy subjects, periodontitis, and dental caries. Contemp Clin Dent.

[9] Hirotomi T, Yoshihara A, Ogawa H, Ito K, Igarashi A, Miyazaki H (2006). A preliminary study on the relationship between stimulated saliva and periodontal conditions in community-dwelling elderly people. J Dent.

[10] Zulkarnain Sinor AA (2013). Association between salivary parameters and periodontal disease. International Medical Journal.

[11] Navazesh M, Kumar SK, University of Southern California School of D (2008). Measuring salivary flow: Challenges and opportunities. J Am Dent Assoc.

[12] Fenoll-Palomares C, Munoz Montagud JV, Sanchiz V, Herreros B, Hernandez V, Minguez M (2004). Unstimulated salivary flow rate, ph and buffer capacity of saliva in healthy volunteers. Rev Esp Enferm Dig.

[13] Sawair FA, Ryalat S, Shayyab M, Saku T (2009). The unstimulated salivary flow rate in a jordanian healthy adult population. J Clin Med Res.

[14] Shimazaki Y, Fu B, Yonemoto K, Akifusa S, Shibata Y, Takeshita T (2017). Stimulated salivary flow rate and oral health status. J Oral Sci.

[15] Gilmore J (1999). Body mass index and health. Health Rep.

[16] Greene JC, Vermillion JR (1964). The simplified oral hygiene index. J Am Dent Assoc.

[17] (2013). Oral health surveys : Basic methods 5th edition.

[18] Ainamo J, Barmes D, Beagrie G, Cutress T, Martin J, Sardo-Infirri J (1982). Development of the world health organization (who) community periodontal index of treatment needs (cpitn). Int Dent J.

[19] Dodds MW, Johnson DA, Yeh CK (2005). Health benefits of saliva: A review. J Dent.

[20] Petrusic N, Posavac M, Sabol I, Mravak-Stipetic M (2015). The effect of tobacco smoking on salivation. Acta Stomatol Croat.

[21] Armitage GC (1999). Development of a classification system for periodontal diseases and conditions. Ann Periodontol.

[22] (1999). The pathogenesis of periodontal diseases. J Periodontol.

[23] Shin YU, Lim HW, Hong EH, Kang MH, Seong M, Nam E (2017). The association between periodontal disease and age-related macular degeneration in the korea national health and nutrition examination survey: A cross-sectional observational study. Medicine (Baltimore).

[24] Idrees MM, Azzeghaiby SN, Hammad MM, Kujan OB (2014). Prevalence and severity of plaque-induced gingivitis in a saudi adult population. Saudi medical journal.

[25] Lagana G, Abazi Y, Beshiri Nastasi E, Vinjolli F, Fabi F, Divizia M (2015). Oral health conditions in an albanian adolescent population: An epidemiological study. BMC Oral Health.

[26] Alcouffe F (1989). Oral hygiene behavior: Differences between men and women. Clin Prev Dent.

[27] Inoue H, Ono K, Masuda W, Morimoto Y, Tanaka T, Yokota M (2006). Gender difference in unstimulated whole saliva flow rate and salivary gland sizes. Archives of oral biology.

[28] Percival RS, Challacombe SJ, Marsh PD (1994). Flow rates of resting whole and stimulated parotid saliva in relation to age and gender. J Dent Res.

[29] Flink H, Bergdahl M, Tegelberg A, Rosenblad A, Lagerlof F (2008). Prevalence of hyposalivation in relation to general health, body mass index and remaining teeth in different age groups of adults. Community Dent Oral Epidemiol.

[30] Leone CW, Oppenheim FG (2001). Physical and chemical aspects of saliva as indicators of risk for dental caries in humans. J Dent Educ.

